# Status and Perspective of High-Energy Beam Surface Strengthening: High-Speed Steel

**DOI:** 10.3390/ma15176129

**Published:** 2022-09-03

**Authors:** Shang Li, Xuanpu Dong, Shuren Guo, Xinwang Liu, Huatang Cao

**Affiliations:** 1 State Key Laboratory of Materials Processing and Die & Mould Technology, School of Materials Science and Engineering, Huazhong University of Science and Technology, Wuhan 430074, China; 2 Fujian Science & Technology Innovation Laboratory for Optoelectronic Information of China, Fuzhou 350108, China

**Keywords:** high-speed steel, high-energy beam processing, surface strengthening, wear, corrosion, coating

## Abstract

High-speed steel (HSS) is primarily used to manufacture cutting tools and roll materials for various machine tools. Improving the hardness, wear resistance, and corrosion resistance of HSS is of great significance to the development of the manufacturing and tool industries. The high-energy beams, consisting of laser, plasma beam, and electron beam processing (e.g., surface remelting, cladding, and alloying), have the advantageous characteristics of high heat source energy and good surface processing effect. The research status and perspective of the above three processing techniques on the surface properties (in particular, hardness, wear resistance, and corrosion resistance) of HSS is reviewed, and the principles, advantages, and disadvantages of the three strengthening methods are discussed. High-energy beam surface alloying appears to be the most cost-effective of HSS surface strengthening methods and is promising to receive increasing research attentions in the future.

## 1. Introduction

High-Speed Steel (HSS) is a high-carbon, high-alloy steel containing significant quantities of W, Mo, Cr, Co, V, etc. HSS was developed in 1898 by American engineers F. W. Taylor and J. M. White at Bethlehem Steel Company [1]. Based on their properties, HSS can be divided into low-alloy high-speed steel (HSS-L), ordinary high-speed steel (HSS), and high-performance high-speed steel (HSS-E), as shown in Table 1 [2]. 

High thermal hardness is the primary performance attribute of HSS. When HSS is cut at high speed, the edge temperature rises to approximately 600 °C and the hardness remains over 55 HRC. Therefore, HSS can keep its high-speed cutting ability and wear resistance at higher temperatures [3], in addition to its adequately high strength, acceptable plasticity and toughness, and high hardenability [4,5,6,7]. Consequently, HSS is widely utilized in the production of various machining tools with large dimensions, fast cutting speeds, heavy loads, and high operating temperatures, such as turning tools, file tools, milling tools, planning tools, broaches, and drills, among others. Additionally, it can be utilized to produce cold and hot dies that require high wear resistance [8,9,10].

The performance requirements for cutting tools and molds have increased in tandem with the performance of processed materials. Therefore, it is necessary to reinforce the material’s surface in order to fulfill the growing processing requirements economically and effectively. It is difficult to match the actual demand by adding element types alone, so it is important to reinforce HSS from a process perspective. The new HSS satisfies the performance demand by altering the elemental compositions. The most common wear-resistant carbide is presumably vanadium carbide, followed by tungsten carbide, molybdenum carbide, alongside chromium carbide and tungsten carbide. The hardest carbides are MC-type carbides. Therefore, vanadium and niobium MC-type carbides are typically used as reinforcing phases in new high-speed steels. Due to the high cost of niobium, high vanadium and high carbon high-speed steels are new advances of modern high-speed steels [11]. For example, CPM10V (CPM-Crucible Particle Metallurgy), W12Cr4V4Mo, W9Cr4V5Co3, and others are shown in Table 1 [12,13,14,15,16,17,18]. In addition to its high wear resistance and toughness, CPM10V steel also possesses excellent toughness and machinability, and is therefore favorably used in the field of cold work tool steel that cannot be solved due to poor wear resistance of other candidates. 

**Figure 1:**
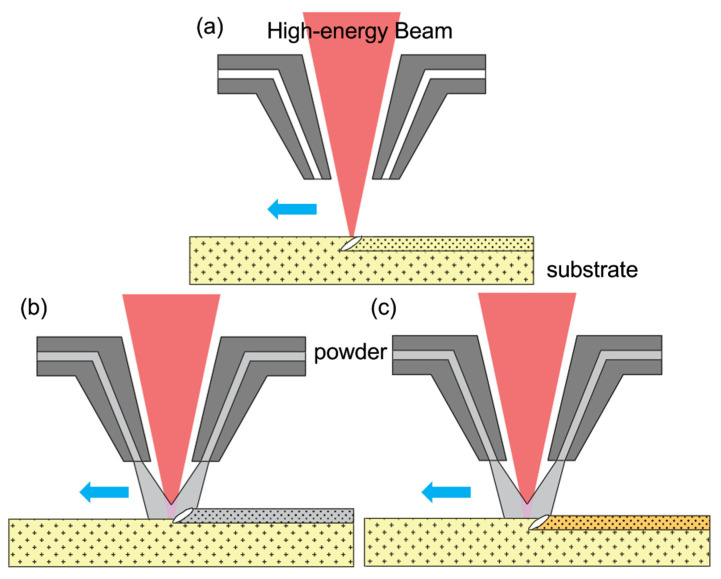
Schematic of processing method: (**a**) High-energy beam remelting; (**b**) High-energy beam cladding; (**c**) High-energy beam alloying.

The use of high-energy beams (e.g., plasma beams, laser beams, and electron beams) to modify the surfaces of materials is a surface modification technique developed in recent decades [19,20,21]. When the high-energy beam acts on the surface of the workpiece, the energy density is extremely high, the heat-affected zone (HAZ) is small, the heating is rapid, the surface of the workpiece is instantly melted and solidified, and quenching can be achieved, and the surface of the workpiece can be locally strengthened without affecting the structure of the workpiece matrix [22,23,24,25,26]. The heat distortion of the workpiece is minimal, and the process is very controllable. Figure 1 shows a schematic illustration of the three reinforcement approaches (high-energy beam remelting, high-energy beam cladding and high-energy beam surface alloying). As illustrated in Figure 1a, high-energy beam remelting does not alter the elemental composition of the substrate because of no exterior addition of other materials. The high-energy beam rapidly melts and cools the substrate’s surface to facilitate the dissolution, ultimately enhancing the substrate’s characteristics. As shown in Figure 1b, high-energy beam cladding only melts the additional metal powder; the substrate scarcely dissolves. The coating composition is newly added metal, and the improvement in surface performance is dependent on the metallic coating. As seen in Figure 1c, high-energy beam alloying simultaneously melts the substrate and adds metal powder. The composition of the coating changes, and the enhanced performance depends on the properties of the new coating metal created by the new metallurgical reaction. High-energy beam remelting, high-energy beam cladding, and high-energy beam surface alloying processing of interests to summarize the recent advancement of HSS surface strengthening treatment from such three perspectives.

## 2. Research Progress on the Enhancement of HSS’s Surface Characteristics by High-Energy Beam Remelting

Typically, the principle of using a laser as a heat source for remelting and strengthening HSS is to use laser scanning to heat the surface of the material, which triggers the surface of the substrate to melt and then rely on the self-excited cooling of the substrate material to condense into a hardened layer. Laser sintering of the workpiece surface can refine the microstructure, reduce surface defects, and enhance wear resistance [21]. Similarly, when the high-energy beam is used as a heat source for rapid heating of the workpiece surface, the temperature of the heated part reaches above the phase transition temperature in a very short amount of time and then relies on the workpiece’s own cooling and phase transition to obtain the desired microstructure, thereby achieving excellent surface properties, including high wear resistance and corrosion resistance [27].

As the laser power density can reach 10^7^ W/cm^2^ and the central temperature of the plasma arc can reach 10^5^–10^6^ K orders of magnitude [28,29], they can be both used as heat sources to scan the heated metal part surface after the substrate melts and cool by self-excited cooling to obtain very fine or ultra-fine grain microstructure and high-hardness carbides. The workpiece’s hardness, wear resistance, corrosion resistance, fatigue resistance, and oxidation resistance are significantly enhanced by the phase hardening layer’s ultrafine grains, exceptionally high dislocation density, and ability to produce compressive stress in the surface layer.

### 2.1. Research Development of High-Energy Beam Remelting on HSS Surface Hardness

In evaluating the mechanical properties of HSS in service, hardness is one of the most primary performance indicators. Throughout the working process, cutting instruments will be subjected to a variety of strains. Therefore, HSS should have adequate hardness to prevent damage during the high-speed cutting, thereby ensuring processing consistency and work productivity. When a high-energy beam is used to treat the surface of a HSS sample, the surface quenching effect is produced by rapid heating and rapid cooling (10^2^~10^6^ °C/s), which results in the dissolution of carbides and an increase in the carbon content of martensite, significantly increasing the surface hardness of the HSS. The effect of hardness enhancement is summarized in Table 2.

For some HSS such as the substrate, high-energy beam remelting can enhance the surface hardness by about 150 HV. The degree of the increased hardness increases with the high-energy beam’s power. For instance, Chen et al. [42] used W18Cr4V HSS as the substrate, and after laser remelting treatment with 1.2~1.5 kW laser, the transition layer had the highest hardness, 950 HV, which was approximately 150 HV higher than the hardness of the substrate W18Cr4V HSS, and the hardness of the transition layer was further improved after tempering treatment at 640 °C. Tuo et al. [43] used an 800 W laser for laser hardening treatment on the surface of HSS rolls and discovered that the hardness after treatment could reach up to 68.5 HRC, whereas the original hardness of the substrate was 59.2 HRC, yielding an improvement of 15.7%, primarily because the carbides became spherical and evenly distributed in the substrate after laser hardening treatment. Li et al. [44,45] strengthened the surface of M2 HSS with a plasma beam, and the hardness of M2 HSS increased by approximately 150 HV in the near-surface layer of approximately 250 μm under the working condition treatment of an acceleration voltage of 200 kV, beam density of 180 A/cm^2^, the pulse width of 150 μs, and a number of pulses of 10 times. When the voltage for electron beam acceleration was increased to 40 kV, the beam current density was 200 A/cm^2^, the pulse width was 5 μs, and there were 9 pulses. The average hardness of M2 HSS has been increased by approximately 118 HV [46]. Chen et al. [47] remelted and strengthened M6 HSS by electron beam, and the average microhardness was 800 HV, which was significantly greater than that of the substrate (283 HV) after spheroidization treatment, consequently the improvement in hardness was evident.

**Figure 2:**
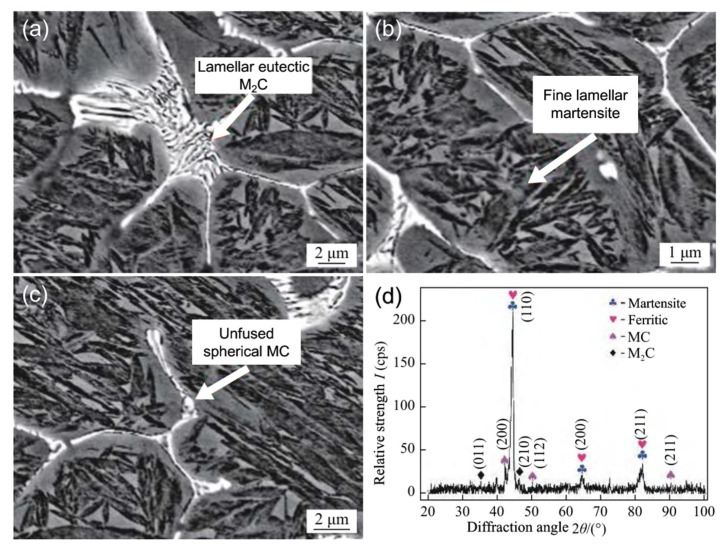
SEM image of W6 HSS (**a**) Lamellar M_2_C eutectic carbide; (**b**) Fine flake martensite; (**c**) Unfused spherical MC carbide; (**d**) XRD results [47].

Figure 2 shows the structure of laser-remelted W6 HSS following remelting. As shown in Figure 1a, there are lamellar eutectic carbides in the intergranular interval, and the results of XRD analysis in Figure 2d show that the lamellar eutectic carbides and the fine rod-like carbides at the end of the eutectic phase are M_2_C (hcp) phase and MC (fcc) phase, respectively. As shown in Figure 2b, a significant amount of acicular martensite was formed within the grains near the remelting zone boundary. As shown in Figure 2c, fine, white, unmelted spherical carbides are present in the middle of the reticulated carbides near the remelting zone boundary. Because of the rapid cooling rate at the boundary during remelting, dissolved carbide cannot disperse, and the melting and solidification process is completed in the original location. In the process of high energy beam remelting, when the high energy beam irradiates the surface of the HSS, the remelting zone undergoes considerable structural changes. The original bulk carbide decomposes and becomes uniformly distributed, the mesh carbide is produced at the grain border, and the needle martensite is produced within the grain (see Figure 2b). In addition, the alloying elements form a complex segregation zone which promotes the development of brittle carbides and reticulated carbides at the grain boundaries, and the hardness of the metal in the remelting zone increases substantially. Adjusting the power and scanning speed of the high-energy beam to control the precipitation of carbide and martensite production in the remelting zone becomes the primary method for increasing the surface hardness of HSS through the high-energy beam remelting process.

### 2.2. High-Energy Beam Remelting Surface to Improve the Wear Resistance of HSS

Wear resistance is the capacity to withstand wear. Due to the high precision and efficiency requirements of HSS tool processing, it is vital to preserving the extremely precise size and surface roughness of the tool. Therefore, wear resistance is crucial for ensuring the cutting quality of its mechanical qualities. High temperature and heat resistance, comprising high temperature hardness and microstructure stability, are the primary determinants of wear resistance. Similarly, the morphology, size, and distribution of carbide in the microstructure have a substantial effect on the wear resistance of HSS. To some extent, the wear resistance of HSS increases proportionally with its hardness. However, once the hardness reaches a certain value, the wear resistance no longer varies with the uplift of hardness, and instead it decreases when the increase in hardness of HSS causes its brittleness [48].

**Figure 3:**
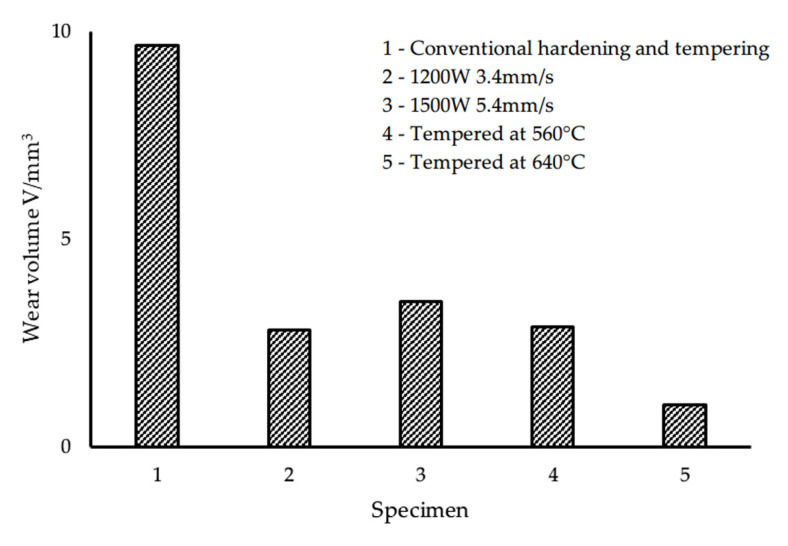
Wear test results of W18Cr4V HSS [42].

**Figure 4:**
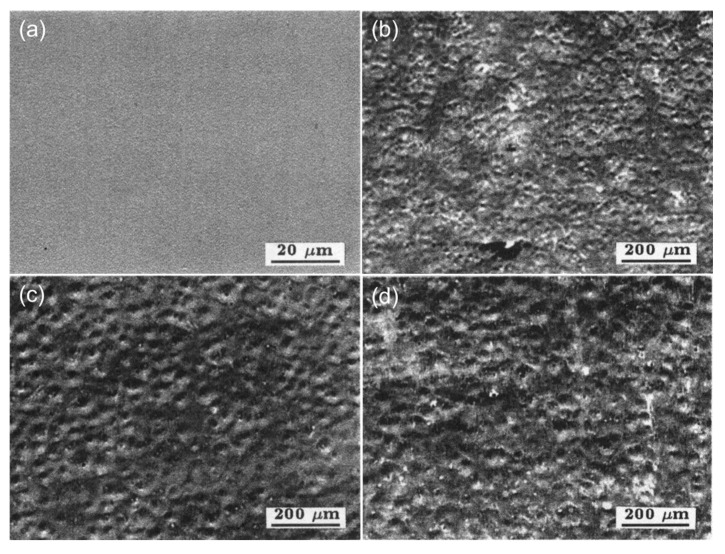
SEM images of the surfaces of HSS (**a**) non-irradiated; (**b**) irradiated with 1 pulse; (**c**) irradiated with 3 pulses; (**d**) irradiated with 5 pulses showing the crater formation after irradiation by HIPIB [49].

The treatment of high energy beam remelting increases the wear resistance of different HSS surfaces by a factor ranging from 2 to 10. Chen et al. [42] employed W18Cr4V HSS as the substrate, and after laser remelting treatment with a power of 1500 W, a scanning speed of 5.4 mm/s, and tempering treatment at 640 °C, the wear resistance of HSS was greatly enhanced. As shown in Figure 3, the wear volume of W18Cr4V HSS treated by laser remelting + tempering at 640 °C was approximately one-tenth that of conventionally quenched and tempered W18Cr4V HSS. Li et al. [45] fortified the surface of M2 HSS with ion beam, and the test wear volume of M2 HSS was reduced from 16 mg to 9 mg under the working condition treatment of accelerating voltage of 200 kV, beam density of 180 A/cm^2^, pulse width of 150 μs, and pulse number 10 times, indicating that the wear resistance of M2 HSS was enhanced by nearly 2 times after ion beam remelting treatment. When Mei et al. [49] modified the working circumstances to an accelerating voltage of 250 kV, a beam current density of 160 A/cm^2^, a pulse width of 80–100 ns, and a pulse count of 5, the authors reached similar conclusions for the treated M2 HSS, but the treated layer was reduced from 250 μm to 200 μm. SEM images of the surface morphologies of HSS before and after the high intensity pulsed ion beam (HIPIB) irradiation treatment are shown in Figure 4. After HIPIB treatment, certain crater-like craters occur on the surface of HSS, as shown in the image. The number of craters continues to diminish as the number of treatments increases, and the craters’ borders become less distinct (see Figure 4b–d). Liao et al. [50] treated the surface of M42 HSS with pulsed explosion plasma and discovered that a pulse count of 2 provided the greatest increase in wear resistance. After treatment, the test wear loss decreased from 2.6 mg to 0.5 mg, indicating that the wear resistance of M42 increased almost 5 times. Yu et al. [51] tested M2 HSS with a pulsed blast-plasma beam and discovered that at 10 pulses, the surface wear performance of M2 HSS was enhanced by 2.3 times.

Quick heating, melting, and rapid cooling generate a new structural layer on the surface of the material during high energy beam remelting, resulting in grain refinement. The refinement of grains prevents the creation and growth of wear cracks, while the transition of *α*-Fe to *γ*-Fe in the treated surface and the generation of a high number of defects [49] such as dislocations enhance the surface wear resistance of the material.

### 2.3. High-Energy Beam Remelting Enhances Corrosion Resistance of HSS Surfaces

The corrosion resistance refers to the capacity to resist corrosion, as HSS is frequently used as a manufacturing tool for use in hostile environments. Therefore, corrosion resistance assures the durability of HSS in extreme service conditions and prevents the inaccurate size, high surface roughness, and other mechanical qualities of HSS-made tools caused by corrosion elements in the environment.

**Figure 5:**
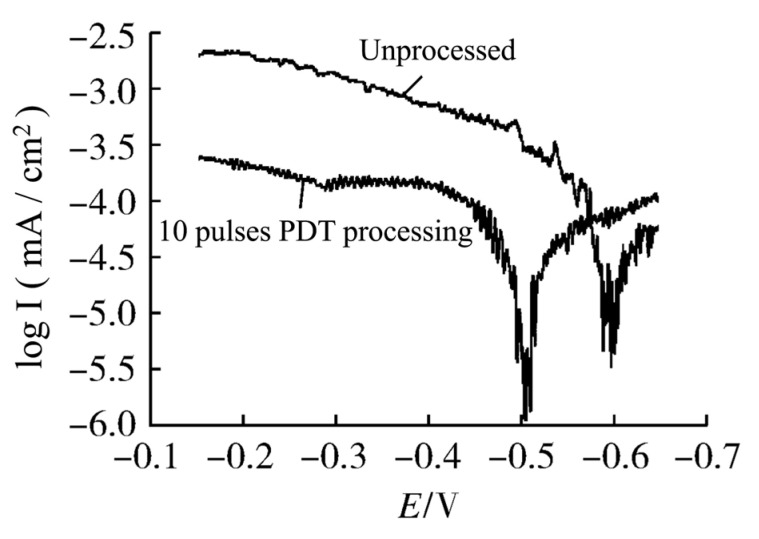
The dynamic potential polarization curves of M2 HSS before and after PDT [51].

Li et al. [46] surface modified M2 HSS with an electron beam acceleration voltage of 40 kV, beam current density of 200 A/cm^2^, pulse width of 5 s, and number of pulses of 9 under working conditions, and then conducted corrosion tests. They discovered that the corrosion increment decreased from 0.0016 g to 0.0011 g, and the corrosion resistance of remelted HSS was significantly enhanced. Yu et al. [51] showed that after 10 pulses of treatment with a pulsed explosion-plasma beam, the corrosion resistance of M2 HSS was greatly enhanced. The dynamic potential polarization curves of M2 HSS before and after plasma detonation treatment (PDT) are shown in Figure 5. After 10 pulses of PDT treatment, the self-corrosion potential of the PDT sample increased from −0.590 V to −0.510 V; the corrosion rate of the PDT sample was 8.27 × 10^−4^ mm/a, and the self-corrosion current density was 3.508 × 10^−5^ mA/cm^2^; the corrosion rate of M2 HSS is 3.81 × 10^−3^ mm/a, and the self-corrosion current density is 1.865 × 10^−4^ mA/cm^2^. It can be seen that the corrosion resistance of M2 HSS is obviously improved after PDT treatment. The surface layer of untreated M2 HSS is predominantly formed of martensite *α*′-Fe and MC (M is mainly W and Cr). The surface of M2 HSS underwent a change from martensite *α*′-Fe to austenite *γ*-Fe with a fcc structure after PDT treatment, which is because the surface carbides of the HSS are dissolved and disseminated during the quick heating process, and during the rapid cooling process, the high-temperature liquid-phase austenite remains at normal temperature and the carbides are solid-dissolved within the interior austenite.

**Figure 6:**
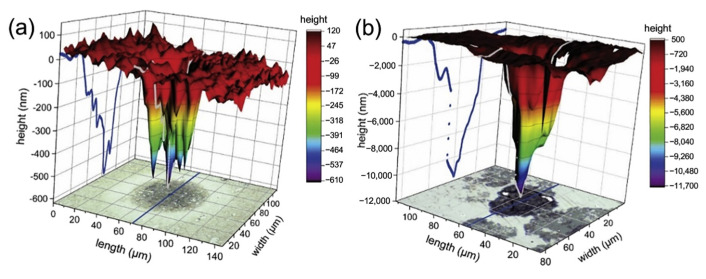
Local reconstruction of M35 HSS pitting corrosion after 1 day (**a**) and after 7 days (**b**) [30].

Pitting corrosion is a common kind of corrosion on metal surfaces. The pitting corrosion is strongly attributed to the material inhomogeneity (casting defects, differences in grain structure, inclusions, gran boundary defects, etc.) on the surface of the metal sample. The predominant cracking initiation caused by pitting corrosion can be correlated to the primary austenite grain boundaries, as these sites are normally weaker in martensitic steels. Furthermore, the formation of carbide chains can also result in faster crack propagation along the incoherent boundaries. Figure 6 shows the final size and shape of the M35 HSS pitting, which expanded from a small, localized pit to a single, huge pit. The majority of the expansion of the pits is vertical, and the main pit shape is elliptical with higher depth. As shown in Equation (1), Matic J K et al. performed a Weibull distribution of the relative size of pits on the distribution and influence of corrosion propagation over time, where β is the shape parameter (Weibull slope) and η is the scale parameter. It was found that metals with a smaller structure and more network structure have a higher corrosion resistance, and the emergence of corrosion pits is slower [30]. Due to the high energy of the heat source of the high-energy beam, the impurities adsorbed on the surface of the HSS evaporated or vaporized, hence reducing the hazards of pitting corrosion and increasing the material’s corrosion resistance. The corrosion resistance of HSS is proportional to the austenitic *γ*-Fe content of its surface layer [31,32]. During tip discharge, the W electrode rod is partially vaporized, and W elements infiltrate the surface of HSS, which increases the material’s corrosion resistance [33,34].

(1) $$f\left(t\right)\equiv \frac{\beta }{\eta }{\left(\frac{t}{\eta }\right)}^{\beta -1}e^{-{\left(\frac{t}{\eta }\right)}^{\beta }}$$

**Figure 7:**
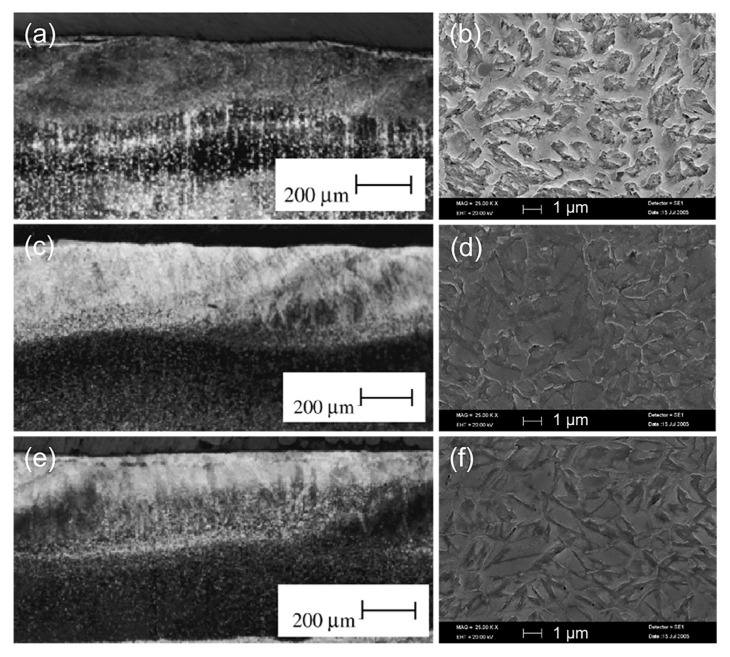
Optical images of cross-sectional appearance and SEM images of the melting zone (MZ)of the laser-melted specimens (**a**,**b**) LSM-M2; (**c**,**d**) LSM-ASP23; (**e**,**f**) LSM-ASP30 [35].

Kwok C T et al. [35] carried out laser remelting on the surfaces of M2, ASP23, and ASP30 HSS, and discovered that the corrosion current density was decreased and the corrosion resistance of HSS was strengthened. The combined effect of refinement of bulky carbides in the HSS microstructure and higher solubility of passivating alloys in austenite and martensite, as a result of laser remelting, enhanced the corrosion resistance of HSS, with ASP-23 HSS exhibiting the highest corrosion resistance after laser remelting. Optical images of cross-sectional appearance and the SEM images of the melting zone (MZ)of the laser-melted specimens (LMS) are shown in Figure 7.

As shown in Figure 7a,c,e, in the regions where successive laser tracks overlapped, the melted surfaces of all specimens were relatively smooth and devoid of fissures. Figure 7b shows the SEM image of the LSM-M2 melting zone. At the interdendritic boundaries, a continuous network of eutectic carbides (*γ–K*) was observed, and the dendrites consisted of *γ* and *α′*. In contrast, LSM-ASP23 and LSM-ASP30 exhibited a more homogeneous and finer dendritic or cellular structure of *γ* and *α′* (Figure 7d,f).

## 3. Research Advancements Concerning the Improvement of the Surface Properties of HSS by High-Energy Beam Cladding

High-energy beam surface cladding technology is an emerging metal surface modification and surface treatment technology that utilizes high-energy beams as a heat source to fuse a coating with wear, heat, and corrosion resistance properties to the surface of a substrate material via rapid melting, expansion, and instantaneous solidification, with cooling rates typically reaching 10^2^~10^6^ °C/s, thus constituting a new composite gradient material. The coating has a high bonding strength, a dense coating structure, low porosity, and few impurities [52]. When the surface is melted by the high-energy beam, only the full precoat is melted, while the surface of the substrate is only partially remelted, preserving the precoat’s composition [53]. Only a thick band of approximately 5 μm at the substrate bond is diluted. Due to HSS’s high hot hardness, wear resistance, and other benefits, researchers use powder or wire containing HSS components to strengthen the base material by cladding, thus altering the surface properties of the base material.

High-energy beam surface cladding is a rapid and stable method for producing HSS coatings of diverse material compositions on the surface of an inferior substrate (e.g., carbon steels or cast irons) by adding featured powder materials, which can drastically alter the substrate’s properties while decreasing the cost of employing precious bulk metals. Currently, surface coating cladding technology for high-energy beams is a popular direction, but there is always room for improvement. Future research will focus on the automation of energy control of high energy beams, the influence of various protective gases on high energy beam surface cladding, the composition of coating materials, and the integrity of process equipment. Some metal elements in HSS have high melting temperatures, therefore utilizing a high-energy beam as a heat source for cladding enables the HSS powder or wire to cover the surface of the substrate more uniformly and efficiently.

### 3.1. High-Energy Beam Cladding Research on Surface Hardness of HSS

**Figure 8:**
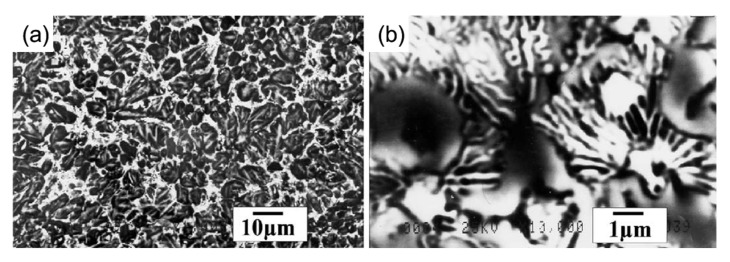
(**a**) SEM micrograph of the alloyed zone. The former austenite grains transformed on cooling to martensite (gray) and are surrounded by globular M_6_C carbides and a eutectic mixture containing M_2_C carbides (white areas); (**b**) SEM showing, in detail, the microstructure of Figure 6a. Dark gray areas are former austenite grains transformed to martensite and are separated by a duplex M_6_C/*γ* and M_2_C/*γ* eutectic. Globular white particles are M_6_C carbides, from which the M_2_C carbides are radially emanating [54].

**Figure 9:**
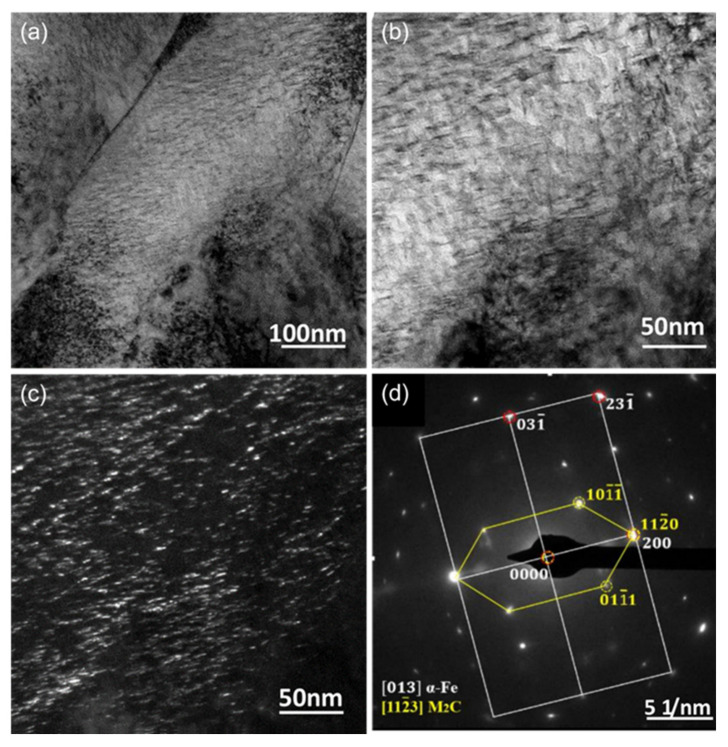
TEM characterization of the C-0.65 HSS coatings after triple-tempering at 530 °C: (**a**,**b**) brightfield images; (**c**) dark field image; (**d**) the corresponding SAED pattern for (**b**) [56].

**Figure 10:**
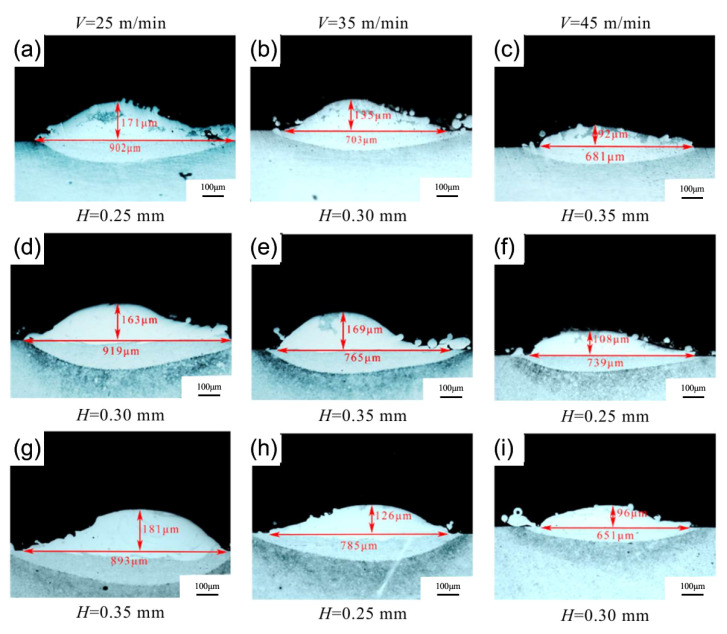
Specimen cross-sectional shape: (**a**–**c**) P = 1500 W; (**d**–**f**) P = 1700 W; (**g**–**i**) P = 1900 W [57].

M2 HSS is a molybdenum-based HSS with minimal carbide inhomogeneity and high toughness. Its powder is frequently used as a raw material to melt and coat other metal surfaces due to its high hardness and wear resistance. There are a huge number of MC, M_2_C, M_6_C, and other compound carbides in the newly produced coating, which significantly boosts its hardness. L. Bourithis et al. [54] synthesized an M2 HSS alloy layer with a homogeneous distribution of alloying elements, a thickness of 1.2–1.5 mm, and a microhardness of 759 HV_0.2_ on carbon steel using a transfer plasma arc (PTA). The alloy layer consisted of martensitic and austenitic dendritic crystals, as well as M_6_C and M_2_C composite carbides, as shown in Figure 8. Zhang et al. [55] clad 966T high hardness wire on M2 HSS base material to provide clad layer microstructure without grain coarsening and with a hardness greater than 60 HRC. Tang et al. [56] fabricated an M2 HSS coating using laser cladding technology; the coating’s solidification microstructure consisted of a martensite matrix, residual austenite, and M_2_C-type carbide; the residual austenite in the coating was essentially eliminated after tempering; and the tempering peak hardness was 813 HV_0.5_. The TEM characterization and the corresponding SAED pattern of the HSS coatings after triple-tempering at 530 °C are shown in Figure 9. As shown in Figure 9a–c, the coating is slate-like martensite after tempering, and the matrix is uniformly dispersed with nanoscale carbides resembling short rods. Figure 9d shows the SAED pattern corresponding to Figure 9b, which displays two sets of diffraction spots at the martensite matrix position, i.e., the slate-martensite and M_2_C phases of the hexagonal crystal structure. The orientation of the nanoscale precipitated carbide precipitated on the martensite matrix is identical to that of the martensite matrix. After triple tempering, the lattice constants a and c of the hexagonal M_2_C phase in the coating fell to 0.2923 nm and 0.4745 nm, respectively, from 0.3011 nm and 0.4771 nm. Zheng et al. [57] determined that laser power of 1900 W, melting speed of 35 m/min, and melting channel spacing of 0.35 mm were the best combinations of parameters for the preparation of M2 coatings by ultra-high-speed laser melting technology, and the prepared M2 clad layer contained a large amount of residual austenite and less carbide content, mainly sub-stable M_2_C and a small amount of MC carbide, so that the coating hardness was high and the coating’s hardness and wear resistance are much superior to those of the matrix. The specimen cross-sectional shapes are shown in Figure 10. Compared to the various cladding speeds, the width of the cladding tracks, and the laser power, it was discovered that as the cladding speed increased, the aspect ratio increased more dramatically and the surface curvature of the single-lane melting layer became smoother. The effect of laser power and the spacing of the cladding tracks on the width and height of the cladding is minimal. The depth of the cladding is significantly affected. The cladding depth increases with the width of the cladding channel and the laser intensity.

**Figure 11:**
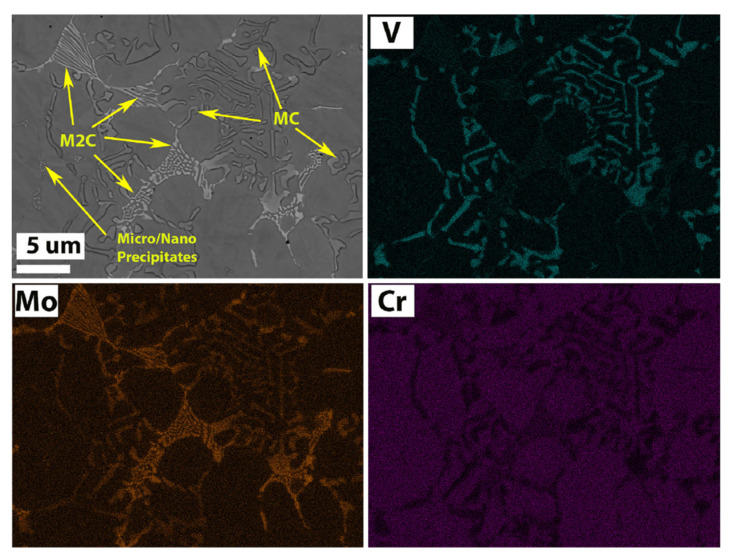
BSE-SEM images showing different carbides present in the laser cladded HSS alloys with EDS images [37].

In addition to M2 HSS, various HSS powders, such as Fe-Co-Mo HSS powder and high vanadium HSS powder, can be utilized to increase the surface hardness of a substrate. Xiong et al. [36] developed Fe-Co-Mo HSS coatings on 40Cr substrates with water-atomized alloy powder as the raw material using the simultaneous powder-feeding laser cladding approach. After post-treatment, the coatings’ hardness could reach 900~950 HV_0.2_. Rahmana et al. [37] laser-melted HSS material onto the surface of 42CrMo4 and discovered that the addition of Co elements to Fe-Cr-Mo-W-V sacrificed Fe (Fe*_bal-x_*-Cr-Mo-W-V-Co_x_), which significantly boosted the coating’s overall hardness. Different carbides present in the laser cladded HSS alloys are shown in Figure 11. EDS maps and a BSE image revealed that MC carbides are rich in V and have blocky, circular, and rod-like shapes (VC). M_2_C carbides have a feathery, lamellar, and layered morphology and are enriched with Mo (Mo_2_C), Mo-containing carbides (Mo_2_C) have a crystalline structure. Fine Cr-enriched secondary precipitates of M_7_C_3_ and M_23_C_6_ are interspersed throughout the matrix.

The technology for melting HSS powder with a high-energy beam consists of melting the existing HSS powder on the surface of the base material. The processed metal covering inherits the hardness characteristics of HSS, while the base material retains its own strength and toughness, so the new metal possesses a variety of good attributes simultaneously and may satisfy more complex needs.

### 3.2. High-Energy Beam Cladding Research on Surface Wear Resistance of HSS

In the high-energy beam melting of HSS coatings, due to the high energy of the high-energy beam, the time required for melting and solidification of HSS powder is very short; as a result, the microstructure grains in the melted HSS coating are fine and not fully grown, thereby achieving the effect of grain refinement. Fine grains and strong carbide particles confer high wear resistance to the HSS coating.

**Figure 12:**
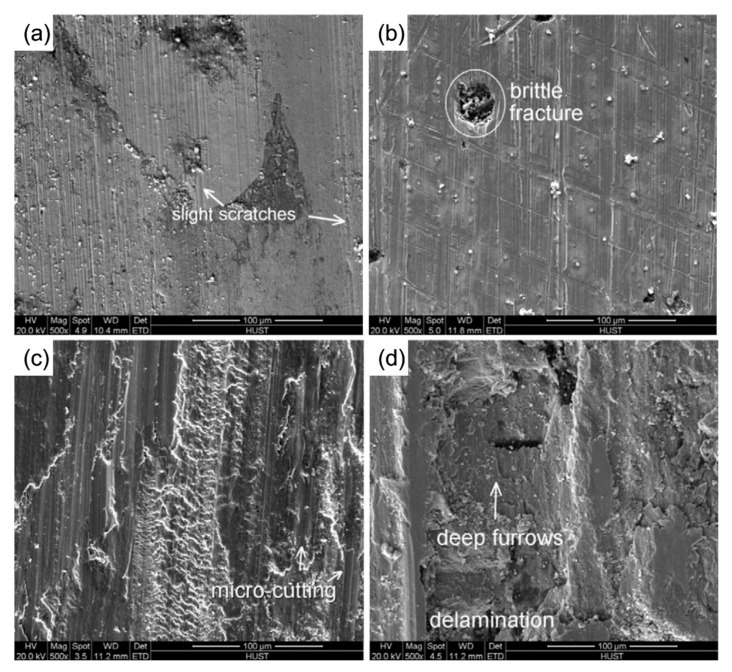
SE-SEM images showing wear morphologies of different samples (200 N): (**a**) PTA-alloyed HVHSS coating; (**b**) PTA-remelted; (**c**) Mn13 steel; (**d**) DI substrate [40].

**Figure 13:**
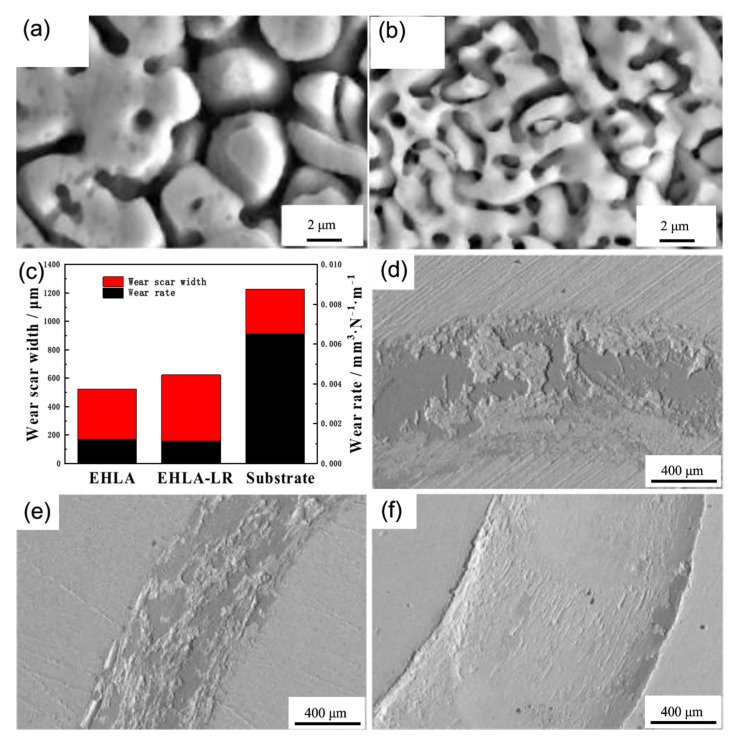
Cross-sectional SEM microstructure in near surface region: (**a**) EHLA; (**b**) EHLA-LR; Wear test results: (**c**) Wear scar width and wear rate; (**d**) Wear appearance of EHLA; (**e**) Wear appearance of EHLA-LR; (**f**) Wear appearance of the substrate [58].

On the surface of the base material, high-energy beam cladding of HSS is utilized, and the hard carbide particles and grain refinement in the coating significantly improve its wear resistance. N. Ur Rahmana et al. [38] discovered that the fine microstructure of laser-coated HSS alloys at 25 °C decreased the contribution of abrasives to wear in comparison to cast HSS. Sun et al. [39] clad austenitic stainless steel’s surface with a molten M3:2 HSS coating, and heat treatment increased the coating’s wear resistance. During dry sliding friction at 500 °C, the maximum wear resistance of the M3:2 HSS coating was attained. Cao et al. [40] fused a high vanadium HVHSS coating onto a ductile cast iron using a high-energy plasma beam; the HVHSS coating exhibited excellent frictional properties due to the mixed hard phases formed by MC, M_7_C_3_, M_23_C_6_, martensite, and grain refinement; and the hard carbide particles acted as a barrier against abrasion and adhesive wear. The wear morphologies of different samples are shown in Figure 12. It clearly shows the wear morphologies of the HVHSS surface are rather shallow and smooth without brittle failures compared to those of remelted duction iron, Mn13 steel, and the iron substrate, respectively. By laser melting and remelting the Fe82Cr16SiB coating, Liu et al. [58] were able to reduce the friction coefficient and improve the wear resistance due to the increased flatness and crystal grain refinement within the coating after the remelting treatment. As shown in Figure 13a,b, the average size of dendrites decreases down from 3~5 μm of the extreme high-speed laser cladded specimen (EHLA) to 1~2 μm of the laser cladded specimen (EHLA-LR). The friction test results of the coating are shown in Figure 13c–f. As shown in Figure 13c, the wear scar width of the extreme high-speed laser remelted specimen (EHLA-LR) is increased, with respect to that of the extreme high-speed laser cladded specimen (EHLA), while the wear rate decreases slightly. Figure 13d–f show the appearance of wear. On the EHLA and EHLA-LR, the substrate has a furrow morphology, indicative of abrasive wear, together with a high amount of spalling.

To further enhance the effect of grain refinement by controlling the energy and action time of the high-energy beam and to increase the proportional content of wear-resistant metals in HSS powder will be the focus of future efforts to improve the wear resistance of HSS surface by high-energy beam melting.

### 3.3. High-Energy Beam Cladding Research on Surface Corrosion Resistance of HSS

In the high energy beam melting of HSS coating, the time required for melting and solidification of HSS powder is very short due to the high energy beam. Therefore, with cladded HSS coating, the microstructure grains are fine and not fully developed, resulting in a grain refining effect. Fine grains and strong carbide particles confer high wear resistance to the HSS coating.

Fang et al. [41] utilized a laser fusion coated Fe-0.5Cr-11Cr alloy coating because to the coating’s finer carbide dispersion and consistent structure. The high Cr content of the coating increased the self and pitting corrosion potentials and decreased the size passivation current density, hence enhancing the material’s corrosion resistance.

## 4. Research Work on the Enhancement of HSS’s Surface Characteristics by High-Energy Beam Surface Alloying

High-energy beam surface alloying is the use of a high-energy beam to melt the metal surface while adding an alloy coating to form a layer of fairly high concentration and fairly uniform alloy layer based on alloying elements as solute and substrate as the solvent, thereby obtaining the required special properties such as wear resistance, corrosion resistance, high temperature resistance, and oxidation resistance, as well as performance beyond the limit of material performance.

The primary distinction between surface alloying and surface cladding is that alloying causes the added alloying elements and a portion of the base material to melt and then undergo a chemical metallurgical reaction in the molten pool; the pre-set coating components are completely diluted (dilution rate = 1). The amount of coating required for surface alloying is only 1/7–1/3 of that required for surface cladding, after industrial requirements are met. It is more efficient and economical. In recent years, it has become an increasingly important research center.

### 4.1. Research Advancements Concerning the Surface Hardness of HSS by Means of High-Energy Beam Surface Alloying

**Figure 14:**
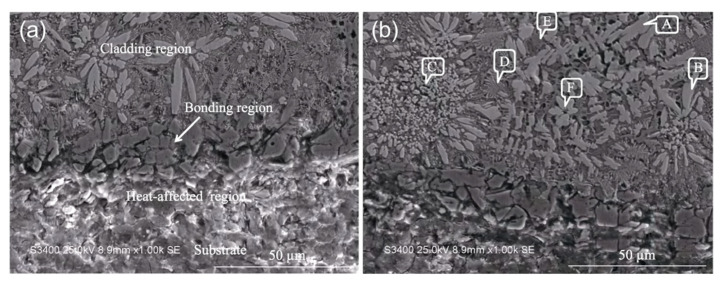
SEM images of bottom region of the cladding layer of sample: (**a**) Cross-section structure of cladding layer; (**b**) Microstructure of cladding layer (A. Fe_3_W_3_C; B. W_2_C, a few Fe_3_W_3_C and Mo_2_V_4_C_5_; C. Fe_3_W_3_C, and WC; D. M_6_C type hard phase with Co element; E. Fe solid solution with a large amount of W and a small amount of Co, C, and other elements; F. Co_6_W_6_C) [60].

**Figure 15:**
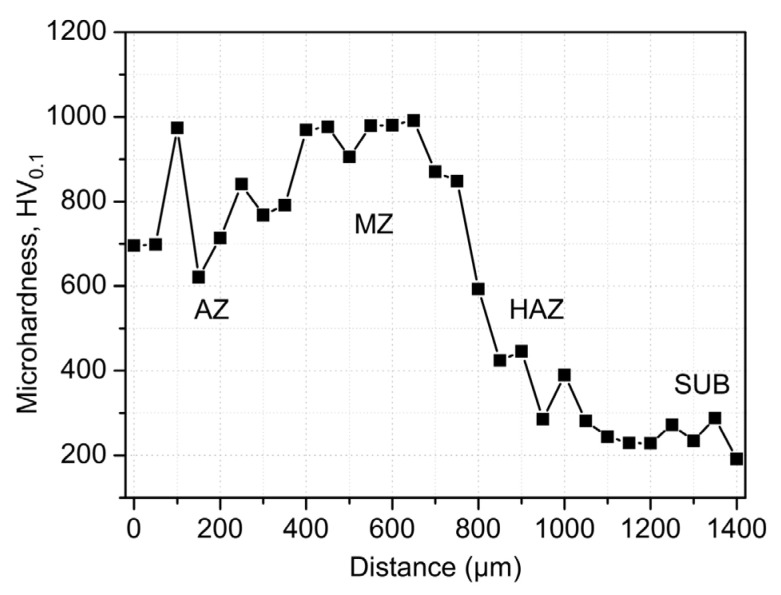
Microhardness profile of HVHSS graded coating on iron substrate as a function of depth [63].

**Figure 16:**
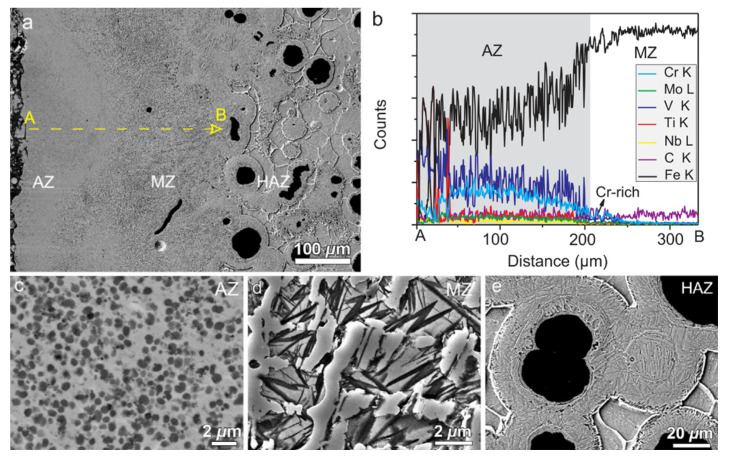
(**a**) SEM images showing the cross-section of the HVHSS graded coating and (**b**) linear EDS scan along the line A–B marked in (**a**); Close view of the microstructure of the alloying zone (AZ) (**c**), melting zone (MZ) (**d**) and heated affected zone (HAZ) (**e**), respectively [64].

**Figure 17:**
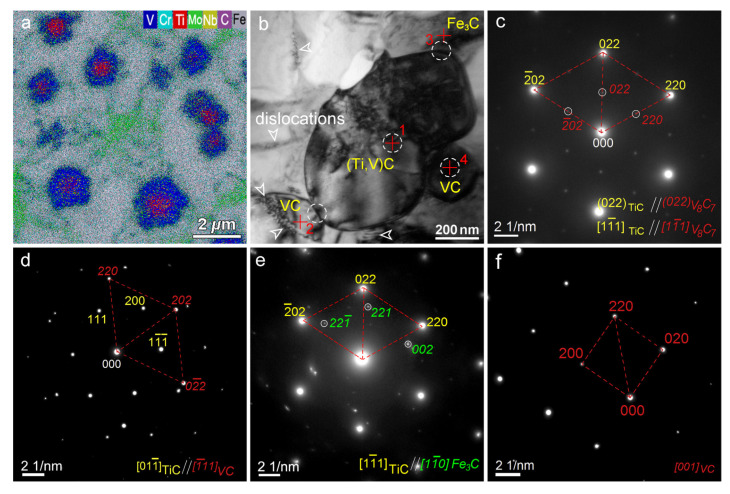
(**a**) EDS area mapping showing TiC/V_8_C_7_ double carbides in a PTA prepared high vanadium HSS coating; (**b**) bright-field TEM image showing MC carbide cluster in the AZ with the crosses numbered for EDS; (**c**–**f**) related SADPs of spot 1–4, respectively, indicated by circles in (**b**) [64].

The metal elements W, V, and Co have high thermal hardness; the hardness of the metal can be significantly increased by pre-placing powder containing these three elements on the surface of HSS and then alloying the surface with a high-energy beam. Under the action of the high-energy beam, the alloying elements in the powder on the surface of the material and the matrix material undergo a metallurgical reaction to form a large number of hardened phases, increasing the hardness of the material. Chen et al. [59,60] melted WC/Co powder on a M2 HSS tool using 1100 W of laser power and 3 mm/s of scanning speed. The average microhardness of the tool’s molten layer reached 1311 HV with many new hard phases, and the average hardness reached 60.24 HRC at 600 °C and still above 50 HRC at 1000 °C. The SEM images of cross section of the alloying layer of sample are shown in Figure 14. From Figure 14a, it can be seen that the cross-sectional structure can be divided into four parts: the cladding region, the bonding region, the heat-affected region, and the substrate. Figure 14b shows that the microstructure is more complex and eutectic, which is mostly the result of the melting pool’s cooling circumstances. Wu et al. [61] fabricated a laser-clad chipbreaker with M2 HSS as the base material and M2/WC-12Co powder, achieving good metallurgical bonding of the materials, no crack flaws in the clad layer, and hardness of up to 907.2 HV. Cao et al. [62,63,64] fused a high-vanadium HVHSS coating onto ductile cast iron using a high-energy plasma beam. The maximum hardness of the coating was 956.5 HV_0.2_, 4.8 times that of the substrate, due to precipitation strengthening of the hard carbide phase, solid solution hardening of the alloying elements, and fine grain hardening due to rapid solidification caused by rapid heating of the plasma beam. Due to the complexation of ultrafine MC and hard phases such as M_2_C, M_7_C_3_, M_23_C_6_, and martensite, the alloying procedure results in a substantial increase in material hardness within a thickness of 800 μm, as shown in Figure 15. It can be seen from Figure 15 that the average microhardness of AZ varies from ~620 HV_0.1_ to 960 HV_0.1_, marginally lower than that of the melting zone MZ. The SEM image, EDS area mapping, TEM images, and SADPs in the coating are shown in Figure 16 and Figure 17, respectively. As can be seen from Figure 16, dense, globular MC carbides are dispersed in the top AZ and substantial amount of needle-like martensite as a hard buffer layer to further support the top AZ to increase the wear life. From Figure 17, the MC type-carbide sever as the nucleation sites for other carbides and the TiC carbide held a crystal orientation relationship with V_8_C_7_. It can be concluded that the graded coating created by the alloying process has very fine MC carbides due to the in-situ formation of exterior strong carbide-forming elements with the C elements from the iron substrate, and the production of a large number of strengthening phases in the coating significantly increases the surface hardness of ductile iron (Figure 15). Figure 16d,e also indicate a strong physical metallurgical bonding between the HVHSS coating with the substrate.

During the alloying process on the high-energy beam’s surface, W generates primarily M_6_C-type carbides in the coating and is the primary component of eutectic carbides. V is predominantly present in the coating as VC, with a tiny amount dissolved in other forms of carbides; V can refine the grain and inhibit the decomposition of martensite, hence enhancing the coating’s hardness. Co’s strong connection with W and Mo atoms retards carbide precipitation and aggregation growth and boosts the alloy’s thermal toughness. However, W, V, and Co can have negative impacts on HSS coatings. Therefore, future research will concentrate on their additive ratios.

### 4.2. Research Advancements Concerning the Surface Wear Resistance of HSS by Means of High-Energy Beam Surface Alloying

The presence of Mo and V in metal elements significantly improves alloys’ resistance to wear. Both components are capable of refining the grain and enhancing the precipitation of hard phases in the alloy, hence enhancing its wear resistance. Consequently, the wear resistance of the metal can be significantly enhanced by alloying the surface of HSS with a high-energy beam and a powder containing Mo and V elements.

**Figure 18:**
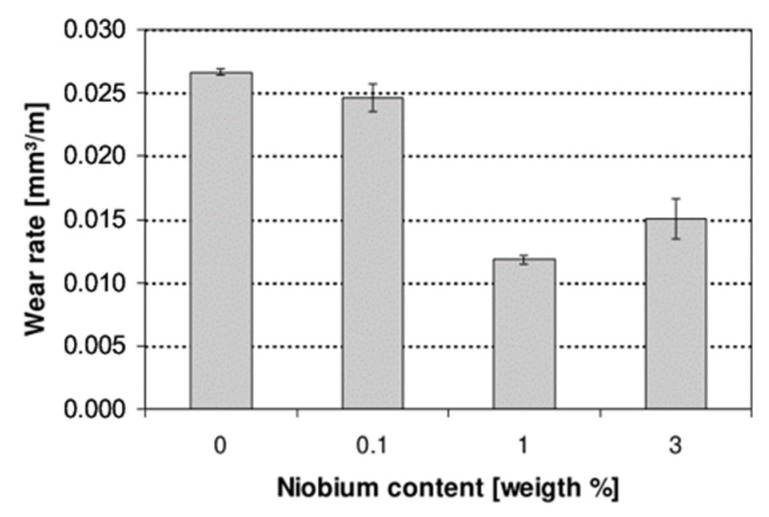
Wear rate for HSS laser hardfacings tested under low-stress abrasion conditions as a function of the niobium content [66].

Wang et al. [65] prepared Ni-based WC alloying on the surface of M2 HSS and discovered that the precipitated graphite and the Ni metal contained within itself have good friction reduction effects, and that the mechanism for friction reduction is the mutual combination of the soft matrix and the hard phase. As illustrated in Figure 18, Ripoll M R et al. [66] laser deposited various amounts of Nb on the surface of HSS and discovered that following elemental alloying, the wear resistance increase of the HSS surface was greatest when the content of Nb was 1 wt.%.

### 4.3. Research Advancements Concerning the Surface Corrosion Resistance of HSS by Means of High-Energy Beam Surface Alloying

The addition of Cr in metal constituents can significantly improve the alloy’s corrosion resistance. In the alloy microstructure, Cr is nearly totally dissolved in austenite, which improves the alloy’s hardenability and boosts its corrosion resistance and oxidation resistance. By adding Cr to the powder of the preset powder, the corrosion resistance of the alloy layer formed after alloying on the surface of the high-energy beam can be enhanced. Due to the high energy of the high-energy beam, which melts and vaporizes the impurities, the resulting alloy layer has a thick structure, which lowers the chance of pitting corrosion.

**Figure 19:**
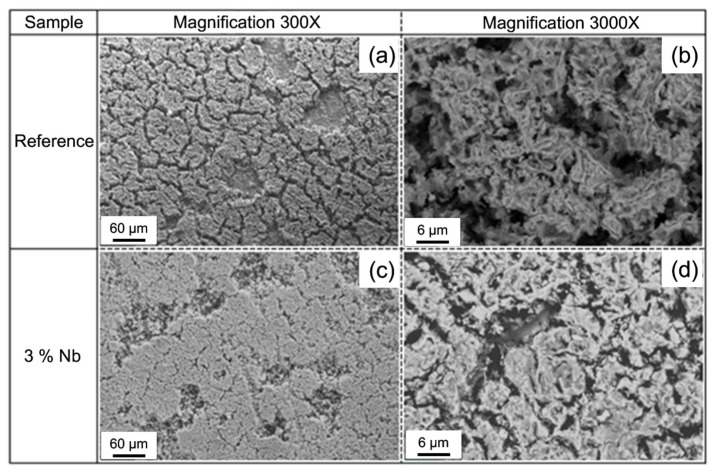
SEM analyses of the corrosion layer present on the HSS laser hardfacings after potentiodynamic tests: (**a**,**b**) the corrosion layer with a porous "mud-cracked" structure; (**c**,**d**) the corrosion layer becomes more continuous and denser after adding 3 % Nb [66].

**Figure 20:**
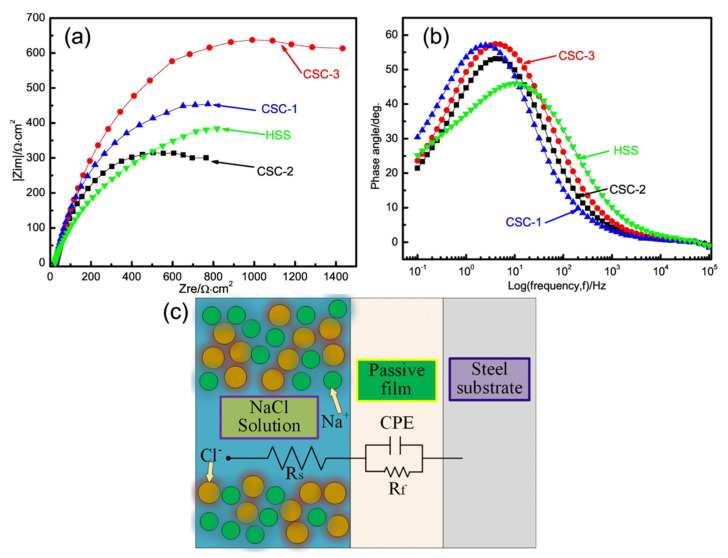
(**a**) Nyquist curves of HSS and different carbon steel claddings; (**b**) phase angle of HSS and different carbon steel claddings; (**c**) Schematic of tested materials [67].

Ripoll et al. [66] deposited various amounts of Nb on the surface of HSS and discovered that following elemental alloying, the best corrosion resistance improvement of the HSS surface was attained when the amount of Nb was 3 wt.%. Nb has a greater affinity for carbon than chromium, and as the Nb percentage rises, less carbon is generated from chromium carbide, therefore less chromium is dissolved in the matrix as an element, thereby enhancing the corrosion resistance of the alloy layer. Figure 19 shows a SEM image of the alloy layer following a corrosion test. The corrosion layer developed on the hard surface of reference was discontinuous and possessed a “mud crack” character (Figure 19a) and has a low density and considerable porosity (see Figure 19b). As the Nb content rises, the scale layer becomes denser, more continuous, and less uncovered by corrosion products (Figure 19c,d). Liu et al. [67] carried out alloying treatment on the surface of carbon steel, and the added materials were HSS and other three kinds of welding wires. Figure 20a,b shows the Nyquist curves of different materials. The impedance spectroscopy exhibits a large semicircular capacitive loop, signifying that the surface film is made of a single layer. The capacitive behavior of surface-modified materials falls in the medium frequency range from 10^0^ to 10^1^ Hz, whereas the medium frequency range of HSS alloys is from 10^1^ to 10^2^ Hz. The HSS alloy peak value is lower than that of carbon steel claddings, indicating the decay of the surface film’s capacitive impedance. Figure 20c shows the equivalent circuit. In this equivalent circuit, R_s_ is the solution resistance; R_f_ represents the passive film’s resistance; the constant phase element (CPE) refers to the film capacitance.

## 5. Summary and Outlook

Since its invention, HSS has expanded swiftly into a wide variety of forms, and is currently undergoing a period of sustained growth. Changing the ratio of metal elements in HSS has limited effect, and it is difficult to match the growing demand. Because of its low material demand and stronger surface requirements, high-energy beam surface strengthening technology has become increasingly attractive for further strengthening HSS surface properties. Due to the fact that it only remelts the surface of HSS substrates, high-energy beam remelting technology offers a moderate performance increase economically and effectively. Furthermore, by utilizing the superior performance of HSS characteristics, high-energy beam cladding technology can be utilized to clad HSS powder onto different metallic substrates, thereby enhancing the overall surface performance of inferior substrates. However, the link between the HSS coating and the substrate is somewhat weak due to the fact that only the packed powder is melted by the melting method. Because the filled powder is melted metallurgically with the base material, high-energy beam surface alloying process consumes less powder and is thus less costly. Additionally, the reinforced layer is more stable and adhesive with the substrate as a result of in-situ formation of metallurgical bonding. Compared to surface remelting, cladding, and alloying, remelting is performed just on the surface of matrix HSS. No new elements are added, and performance is enhanced by altering the structure. During the cladding process, the coating is a newly added material, the matrix barely melts, and the degree of reinforcement depends on the newly added material. The surface alloying is in between; a portion of the matrix and the extra powder are simultaneously melted, and the metallurgical process is carried out. The degree of strengthening is dependent on the quality of the matrix and the addition of powder to regenerate the material by metallurgy. Therefore, high-energy beam surface alloying process will be a potential strengthening technology for HSS-related materials in the near future.

## Figures and Tables

**Table 1:** The classification and trademarks of HSS [2].

Classification	Trademark in China	Trademark in America	Trademark in German	ISO 4957:1999
HSS-L	W3Mo3Cr4V2	—	—	HS3-3-2
	W3Mo3Cr4VSi	—	—	—
HSS	W18Cr4V	T1	1.3355	HS18-0-1
	W2Mo8Cr4V	M1	1.3327	HS1-8-1
	W2Mo9Cr4V2	M7	1.3348	HS2-9-2
	W6Mo5Cr4V2	M2	1.3343	HS6-5-2
	CW6Mo5Cr4V2	CM2		HS6-5-2C
	W6Mo6Cr4V2	M3:1	1.3350	HS6-6-2
	W9Mo3Cr4V	—	—	—
HSS-E	W6Mo6Cr4V3	—	—	HS6-5-3
	CW6Mo6Cr4V3	—	—	HS6-5-3C
	W6Mo6Cr4V4	—	—	HS6-5-4
	W6Mo6Cr4V2Al	—	—	—
	W12Cr4V5Co5	T15	1.3202	—
	W6Mo5Cr4V2Co5	M35	1.3243	HS6-5-2-5
	W6Mo5Cr4V3Co8	M3:2 + Co	1.3244	HS6-5-3-8
	W7Mo4Cr4V2Co5	M41	—	—
	W2Mo9Cr4VCo8	M42	1.3247	HS2-9-1-8
	W10Mo4Cr4V3Co10	M48	1.3207	HS10-4-3-10
	CPM10V			
	W12Cr4V4Mo	EV4		
	W6Mo5Cr4V2C	M36		
	W9Cr4V5Co3			
	W6Mo5Cr4V2Al	M2-Al		
	W12Cr4V3Mo3Co5Si			
	W6Mo5Cr4V5SiNbAl			
	W10Mo4Cr4V3Al			

**Table 2:** Hardness enhancement effects of different intensification processes.

Substrates	Coating Materials Powder	Intensification Process	Hardness	Hardness Enhancement	Refs.
W18Cr4V HSS	—	laser remelting	950 HV	150 HV	[23]
AISID2 tool steel	—	laser remelting	69 HRC	9 HRC	[24]
M2 HSS	—	plasma beam remelting	—	150 HV	[25,26]
		plasma beam remelting	—	115 HV	[27]
		electron beam remelting	800 HV	517 HV	[28]
carbon steel	M2 HSS	transfer plasma arc	759 HV_200_	—	[30]
M2 HSS	966T high hardness wire	laser cladding	60 HRC	—	[31]
Q235 steel	M2 HSS	laser cladding	813 HV_0.5_	—	[32]
9Cr2Mo steel	M2 HSS	laser cladding	800 HV	—	[33]
40Cr steel	Fe-Cr-Mo-W-V HSS	laser cladding	900~950 HV_0.2_	—	[34]
42CrMo_4_ HSS	Fe-Cr-Mo-W-V HSS	laser cladding	843 HV	—	[35]
M2 HSS	WC/Co	laser surface alloying	1311 HV	—	[36,37]
M2 HSS	M2/WC-12Co	laser surface alloying	907 HV	—	[38]
ductile cast iron	HVHSS	plasma beam surface alloying	957 HV_0.2_	757 HV_0.2_	[39,40,41]

## Data Availability

Not applicable.
